# Local Cisplatin Delivery in Mouse Reliably Models Sensorineural Ototoxicity Without Systemic Adverse Effects

**DOI:** 10.3389/fncel.2021.701783

**Published:** 2021-07-14

**Authors:** German Nacher-Soler, Sébastien Lenglet, Marta Coelho, Aurélien Thomas, François Voruz, Karl-Heinz Krause, Pascal Senn, Francis Rousset

**Affiliations:** ^1^The Inner Ear and Olfaction Lab, Department of Pathology and Immunology, Faculty of Medicine, University of Geneva, Geneva, Switzerland; ^2^Forensic Toxicology and Chemistry Unit, University Centre for Legal Medicine, Geneva University Hospital, Geneva, Switzerland; ^3^Faculty Unit of Toxicology, University Centre of Legal Medicine, Lausanne University Hospital, Lausanne, Switzerland; ^4^Department of Clinical Neurosciences, Service of ORL & Head and Neck Surgery, University Hospital of Geneva, Geneva, Switzerland; ^5^Department of Pathology and Immunology, Faculty of Medicine, University of Geneva, Geneva, Switzerland

**Keywords:** refinement, animal welfare, middle ear delivery, cisplatin ototoxicity, cochlea, mass spectrometry

## Abstract

Cisplatin is a lifesaving chemotherapeutic drug with marked ototoxic adverse effects. Cisplatin-induced hearing loss affects a significant part of cancer-surviving patients and is an unmet clinical need with important socioeconomic consequences. Unfortunately, in current preclinical animal models of cisplatin ototoxicity, which are mainly based on systemic delivery, important morbidity is observed, leading to premature death. This methodology not only raises obvious animal welfare concerns but also increases the number of animals used in ototoxicity studies to compensate for dropouts related to early death. To overcome these important limitations, we developed a local delivery model based on the application of a cisplatin solution directly into the otic bulla through a retroauricular approach. The local delivery model reliably induced significant hearing loss with a mean threshold shift ranging from 10 to 30 dB, strongly affecting the high frequencies (22 and 32 kHz). Importantly, mice did not show visible stress or distress indicators and no significant morbidity in comparison with a traditional systemic delivery control group of mice injected intraperitoneally with 10 mg/kg cisplatin, where significant weight loss >10% in all treated animals (without any recovery) led to premature abortion of experiments on day 3. Mass spectrometry confirmed the absence of relevant systemic uptake after local delivery, with platinum accumulation restricted to the cochlea, whereas important platinum concentrations were detected in the liver and kidney of the systemic cisplatin group. A clear correlation between the cochlear platinum concentration and the auditory threshold shift was observed. Immunohistochemistry revealed statistically significant loss of outer hair cells in the basal and apical turns of the cochlea and an important and statistically significant loss of auditory neurons and synapses in all cochlear regions. In conclusion, local cisplatin delivery induces robust hearing loss with minimal morbidity, thereby offering a reliable rodent model for human cisplatin ototoxicity, reducing the number of animals required and showing improved animal welfare compared with traditional systemic models.

## Introduction

Hearing loss (HL) is the most common neurosensory deficit in humans, with over 5% of the worldwide population affected (WHO, March 2020 report), recognized as a major and growing socioeconomic burden leading to cognitive decline, depression, and social isolation (Panza et al., 2015; Rutherford et al., 2018).

Cisplatin (*cis*-diaminedichloroplatinum) is a widely used lifesaving chemotherapy used against different types of cancers, including carcinomas, germ cell tumors, lymphomas, and sarcomas (Dasari and Tchounwou, 2014). Cisplatin harbors antitumor effect through DNA alkylation, interfering with DNA replication and repair mechanisms, ultimately leading to apoptosis in proliferating cells (Basu and Krishnamurthy, 2010). Therefore, the antitumor effect of cisplatin is largely unspecific, leading to important systemic adverse effects including nephrotoxicity (Miller et al., 2010), neurotoxicity, myelosuppression, and ototoxicity (Dasari and Tchounwou, 2014). Cisplatin-related ototoxicity is characterized by irreversible damage to the cochlea leading to permanent sensorineural HL affecting primarily high frequencies, often in combination with tinnitus (Mujica-Mota et al., 2013). The incidence and severity of cisplatin-induced ototoxicity in humans vary widely in the literature, ranging from 12.5 to 100% in children and 26–100% in adults, depending on the HL criteria used for analysis (Landier, 2016). In a retrospective analysis of a large collection of 401 adults treated with cisplatin in Geneva’s University Hospital, significant HL was found in 20% of cases, among whom 60% experienced tinnitus.

Nowadays, no preventive treatment is available to limit the adverse effects of cisplatin on hearing, and its related ototoxicity remains an unmet clinical need, although it is the only clinically relevant acute hearing insult that is foreseeable. Cisplatin ototoxicity conceptually offers a unique window for preventive HL treatment before or simultaneous to the administration of the chemotherapeutic. Addressing and potentially preventing cisplatin ototoxicity requires a deeper understanding of its molecular consequences on the cochlea. However, so far, the development of novel therapies is limited by the lack of a robust and 3R (replace, reduce, and refine principles) compatible preclinical model. The mouse is the most common mammalian model specie, combining short generation time, easy breeding, and relatively low housing costs. Furthermore, the mouse model allows in-depth characterization of putative target genes, as numerous knockout animals are available. Unfortunately, cisplatin HL is difficult to reproduce in a mouse model, as important systemic toxicity generally arises before the onset of mild to moderate ototoxicity. Current mouse models to study ototoxic adverse effects of cisplatin consist of intraperitoneal (IP) cisplatin injection of a single high dose (e.g., Parham, 2011; Naples et al., 2020) or lower doses in a repeated fashion (Breglio et al., 2017; Fernandez et al., 2019; DeBacker et al., 2020). However, in such models, strong toxicity and animal distress are observed, including important weight loss and ataxia, raising important and prohibitive ethical concerns. Because of systemic toxicity and morbidity, many animals are sacrificed before obtaining valid and reproducible results, also limiting the scientific value of the model. Recently, a more clinically relevant model was described consisting of several cycles of serial low doses of cisplatin injections with intercalated resting periods, for a total of 40 days (Breglio et al., 2017; Fernandez et al., 2019). In these studies, the cisplatin treatment was accompanied by a supplementary high-calorie diet and extensive care such as daily injection of saline to overcome the kidney toxicity. This model was reported to produce robust HL; however, it also led to important weight loss (20–30% compared with untreated controls of the same age), morbidity, and costs.

To address the limitations mentioned earlier, we set out to develop a robust and scientifically relevant HL model with minimal morbidity and mortality, in line with 3R principles based on local delivery of cisplatin into the middle ear *via* retroauricular access to the otic bulla. The surgical approach, inspired by previous studies (Stevens et al., 2015; Mulazimoglu et al., 2017), was atraumatic on mouse hearing and did not lead to any signs of morbidity or stress on operated animals. More importantly, this method led to a robust increase of hearing thresholds in cisplatin-delivered ears. Mass spectrometry data showed important accumulation of platinum (Pt) in the operated ear but not in other organs such as the kidney, liver, or in the contralateral ear, which could be safely used as an internal control for hearing threshold comparison and histology. With this minimally invasive method, we also demonstrated a strong correlation between the Pt concentration in the cochlea and the extent of HL. This new preclinical model will not only improve animal welfare and experiment reproducibility but will also provide a model to study long-term consequences of cisplatin exposure on the hearing system, as mice will be able to survive longer and in better condition. We believe that the model is a timely and relevant development, as new and promising otoprotective compounds and preventive strategies are waiting to be tested in preclinical trials.

## Materials and Methods

The main aim of the study is to develop a robust and scientifically relevant HL model with minimal morbidity and mortality, in line with the 3R principles. To do so, a strict animal morbidity evaluation was conducted, comparing the new model with a currently used systemic approach. Secondly, we aim to describe the cisplatin correlation with HL using mass spectrometry analysis. The described protocols were executed in compliance with the Swiss regulations and the Animal Research: Reporting of *in vivo* Experiments guidelines.

### Animal Experimentation

A total of *n* = 52 male and female C57BL/6 mice were used in this study. To avoid age-related HL interferences with the results, the experiments were carried out on young adult mice aged 6 to 8 weeks. The complete research protocol was approved by the local veterinary office and the Commission for Animal Experimentation of the Canton of Geneva (“Commission cantonale pour les expériences sur les animaux”), Switzerland, authorization number GE/149/18.

Animals were blindly assigned into saline 0.9% (control), local cisplatin delivery (5 mM), and IP systemic injection (10 mg/kg) groups, ensuring equal sex distribution. Cisplatin reagent was prepared after resuspension of *cis*-diamineplatinum (II) dichloride (Sigma-Aldrich, 479306) in saline 0.9%, for a final concentration of 5 mM. Animals assigned to the systemic delivery group received a single IP injection of cisplatin (10 mg/kg). As a comparison, systemically injected animals received approximately 0.210 mg of cisplatin (considering an average weight of 21 g), whereas the local delivery group (on average) received 0.012 mg of cisplatin locally.

Animals were anesthetized by an IP injection (dose of 10 μl/g) of ketamine (10%) and xylazine (5%) for the auditory testing and for the surgical procedure. If necessary, 10% ketamine solution was injected intramuscularly (dose 5 μl/g) to elongate the anesthesia and surgery time. During both procedures, the depth of anesthesia was checked every 30 min by testing the pedal withdrawal reflex. Additionally, analgesia was applied during and after the surgery by local injection (1.2 μl/g) of lidocaine and epinephrine (0.5%) to decrease post-surgery discomfort. The animal condition was monitored daily until day 7, recording an animal welfare score sheet.

At the day 7- or 14-time point after local delivery, animals were sacrificed by cervical dislocation under ketamine–xylazine anesthesia followed by decapitation to proceed with the histological and mass spectrometry protocols. Although similar time points were planned for animals receiving cisplatin systemically, the experiment was aborted at day 3, following humane endpoint (15% loss of weight).

### Animal Care Score Sheet

Following the 3R principles, the discomfort and morbidity evaluation was assessed by the design and recording of a score sheet for each experimental subject. The score sheet includes the most relevant variables on rodent welfare, such as weight (general health indicator), piloerection, ataxia, wound condition (infection and sutures condition), and behavior (reaction to manipulation and social interaction). The scoring system was set from 0 (normal condition) to 3 (high severity). Level 1 animals were routinely evaluated, whereas level 2 subjects received analgesia and were closely monitored. Level 3 animals were sacrificed according to Swiss animal welfare legislation. Animal weight and behavior were monitored and evaluated every day, from treatment to death.

### Retroauricular Surgical Approach

**FIGURE 1:**
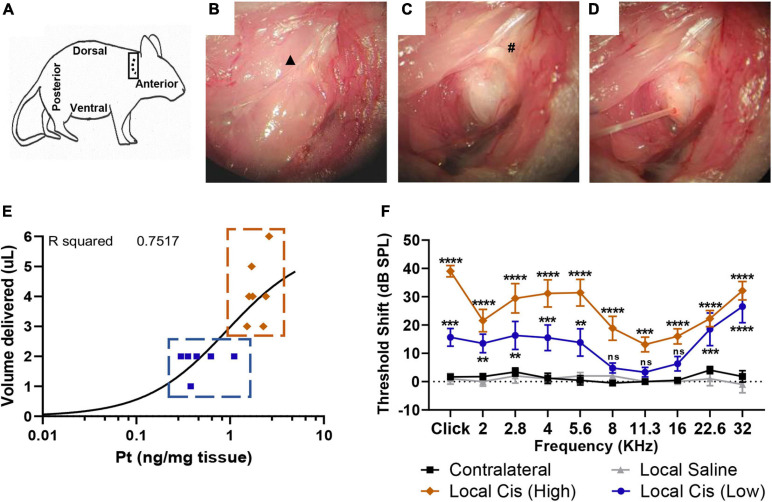
Minimally invasive, retroauricular approach for local cisplatin delivery. To access retroauricular position of bulla, mouse is placed in lateral position, with right ear upward. An incision is made retroauricularly **(A)**. After skin retraction, cervical trapezius muscle and great auricular nerve (▲) are exposed **(B)**. After careful dissection of connective tissue, bulla bone and facial nerve (**#**) are identified **(C)**. Access hole is drilled in a posterior-dorsal position, minimizing risk for cochlear damage. A 0.3-mm diameter cannula is placed into bulla aperture **(D)** to deliver 2–5 μl of solution. Finally, a piece of tissue and cyanoacrylate glue are used to occlude the opening. **(E)** Correlation plot (*n* = 13) between cisplatin (5 mM) volume delivered (limited by bulla size) and Pt fixation. Animals with a volume of cisplatin <3 μl were classified in “low cisplatin” subgroup (blue), whereas animals with a volume of cisplatin ≥3 μl were classified in “high cisplatin” subgroup (orange). **(F)** Permanent threshold shift, measured by unilateral auditory brainstem response (ABR), in cisplatin-treated animals (5 mM, *n* = 13) was recorded 7 days after local delivery in comparison with delivery of saline solution (*n* = 5) and contralateral untreated ears. Cisplatin treated animal data were represented following “high” (orange) and “low” (blue) subclassification defined on **(E)**. The confidence interval for both statistics was set as 95% (*a* = 0.05); ^∗∗^*p* < 0.01; ^∗∗∗^*p* < 0.005; ^****^p < 0.0005.

All surgeries were performed on the animal right ear with a Wild Heerbrug laparoscopy microscope (M655), maintaining the left ear (contralateral) as an internal control. Each animal underwent surgery and treatment only once. Animals were anesthetized, and the surgical procedure started once the proper anesthesia depth was achieved (after the stapedial reflex) and after local analgesia. The animal was placed upon a heating pad, covered with a sterilized cover, in a lateral position allowing access to the right ear. The surgical area was sterilized with 70% alcohol, and the fur was locally shaved with a scalpel. We proceeded with a retroauricular skin incision behind the ear canal (Figure 1A) to access the middle ear of the mice (Figure 1B). The overlying fascia was carefully dissected, allowing us to identify the great auricular nerve, which was used as a reference to identify and retract the cervical trapezius muscle. After the retraction, the bulla bone is identified as located under the facial nerve (Figure 1C). The remaining tissue in the bulla surface was set aside to localize the injection site within the bulla. The bulla was drilled in a posterior-dorsal position to avoid any potential damage during the procedure, using a 25G needle (Figure 1D). Our surgical design allowed the direct contact of the tested solutions with the cochlear round window, facilitating the diffusion into the cochlea. A 20-μl GELoader tip (Eppendorf) was used to deliver the experimental solution through the bulla opening. During 15 min, the bulla was refilled eight times with cisplatin 5 mM or saline 0.9%, maintaining a constant volume of solution into the middle ear, compensating the Eustachian tube drainage. After each 2-μl refill, the solution overflow was removed with a sterilized synthetic surgical sponge (FST, 18105-03).

At the end of the treatment protocol, the bulla space was rinsed three times with 3 μl of saline 0.9% and dried with a synthetic surgical sponge to remove the tested experimental solution from the middle ear. Then, a piece of tissue was disposed of in the bulla opening and sealed with cyanoacrylate glue. Finally, the retroauricular incision was closed by a skin suture. Analgesia was applied locally to reduce post-surgery discomfort. At the end of the surgery, the mice rested upon a heating pad until full recovery.

### Auditory Testing

Animals were anesthetized and placed upon a heating pad to maintain body temperature. All subjects were auditory tested at two time points, the day before the surgery (D0) and 7 (D7) or 14 days (D14) after the local delivery procedure, to measure hearing thresholds. Animals receiving cisplatin systemically were tested at D0 and 3 days after the injection (D3). Auditory brainstem response (ABR) measurements were conducted in a soundproof chamber (IAC Acoustics, IL, United States). Pt electrodes were placed subcutaneously on the forehead of the mouse (+), on the mastoid of the recorded ear (−), and the reference electrode on the back of the mouse. ABRs were recorded after unilateral stimulation through a custom-made earplug adapter with 100-μs clicks or 3-ms tone pipes (2.0 to 32 kHz at a resolution of two steps per octave). The electrical wave outcome was averaged over 128 stimulus repetitions. For the stimulus generation and recording of responses, a multifunction IO-Card (National Instruments, Austin, TX, United States) was used. The sound pressure level (SPL) was controlled with an attenuator and amplifier (Otoconsult, Frankfurt, Germany). Furthermore, the sound pressure was calibrated online before each measurement with a microphone probe system (Bruel&Kjaer 4191) placed near the animals’ ears. Recorded signals were amplified and bandpass filtered (80 dB; 0.2–3.0 kHz) using a filter/amplifier unit (Otoconsult, Frankfurt, Germany). For all frequencies, ABRs were analyzed from 0 to 80 dB SPL in 5 dB steps. The hearing threshold was visually defined as the lowest SPL showing a conserved wave pattern. At the end of the ABR recording, mice were placed in a recovery cage upon a heating pad until consciousness recover.

Threshold shifts were determined as the difference between the post (D7) and presurgery (D0) ABR hearing threshold, providing paired values for all analyzed frequencies in each animal.

### Cochlea Dissection and Histology

At the end of the experiment (D7 ABR), mice were sacrificed for cochlear histology. After the sagittal cut of the skull, the brain and brainstem were removed, and both temporal bones were immersed in 2.5 ml of ice-cold Hanks’ balanced salt solution in a 35-mm Petri dish. The cochleas were extracted under a stereomicroscope (Nikkon, Japan). The dissection procedure allowed the evaluation of the inner ear and middle ear integrity, permitting the evaluation of any possible mechanical damages induced by the surgical approach.

For Pt titration by mass spectrometry, cochleas were weighed on a precision scale, frozen on liquid nitrogen, and conserved at −80°C until analysis. For histology (cytocochleograms and mid-modiolar cuts), cochleas were fixed in 4% formol overnight at room temperature and decalcified for 48 h under sonication (ultrasonic USE 33 decalcification machine, Medite, 03-3300-00) in USEDECALC solution (Medite commercial solution). Finally, decalcified cochleas were microdissected to perform cytocochleograms and immunostaining or embedded in paraffin for mid-modiolar cuts and hematoxylin–eosin staining.

### Mass Spectrometry Analysis

Non-decalcified cochleas (conserved at −80°C) were used on the mass spectrometry analysis. Additionally, to evaluate the cisplatin systemic toxicity, kidney and liver samples were collected as representative tissues. The Pt concentration (LabKings, Hilversum, Netherlands) was measured in the collected mouse tissues (cochlea, liver, and kidney) by inductively coupled plasma mass spectrometry (ICP-MS 7800 series; Agilent, Tokyo, Japan). As previously described (Vorobiev et al., 2019), before analysis, the mouse tissues were weighed and mineralized in Suprapur nitric acid (HNO_3_) 65% (Merck, Darmstadt, Germany) at 80°C for 1 h. The samples, blanks, and calibration curves were diluted in a solution containing HNO_3_ 2%, butanol 1% (VWR, Fontenay-sous-Bois, France), and Triton 0.5% (Sigma, Buchs, Switzerland) in ultrapure water (resistivity greater than 18.2 MΩ cm) (Vorobiev et al., 2019). An external calibration curve in HNO_3_ 65% was prepared by monitoring the 195 Pt isotope with a dwell time of 300 ms in no-gas mode (without collision cell). Internal standards (rhodium and indium; Agilent, Basel, Switzerland) were added at 10 μg/L to the analytical calibration solutions, analytical blanks, and samples. The raw data were acquired using MassHunter software (Agilent, Tokyo, Japan).

### Immunohistochemistry and Microscopy

Decalcified cochleas were microdissected under a binocular microscope (Nikkon, Japan). The bony cochlear wall was removed to uncover the auditory sensory epithelium (organ of Corti), which was separated from the stria vascularis and modiolus. The organ of Corti was divided then into three main sections, the basal, medial, and apical turns, and mounted as cytocochleograms.

The collected organs of Corti were permeabilized [3% Triton-X 100 in phosphate-buffered saline (PBS) 1×] for 30 min at room temperature. After three washing steps with PBS, the samples were placed in blocking solution (2% bovine serum albumin, 0.01% Triton-X 100, in PBS) for 30 min and posteriorly incubated with primary antibodies against Myosin 7a (polyclonal rabbit anti-Myosin 7a, Proteus) and CtBP2 (monoclonal mouse anti-CtBP2, BD Bioscience) (1:200 in blocking solution). Primary antibodies were incubated overnight at 4°C with a 10-rpm agitation on a gyratory rocker (Stuart SSM3). The following day, samples were rinsed three times with PBS and incubated with the secondary antibodies (goat anti-mouse Alexa 555, goat anti-rabbit 488, Life Technologies) (1:500 in blocking solution) for 2 h with a 10-rpm agitation at room temperature. Afterward, samples were washed three times with PBS, placed in glass slides, and mounted with Fluoroshield reagent containing 4′,6-diamidino-2-phenylindole (Sigma).

The image acquisition was performed with a confocal laser-scanning microscope (Zeiss LSM700), using a Plan-Neofluar 20X/0.50 and a Plan-Apochromat 63X/1.4 (Oil) objectives. Images were recorded with a CCD camera (Leica Microsystems) and analyzed with the open-source software FIJI (ImageJ).

### Mid-Modiolar Cuts and Hematoxylin–Eosin Staining

Decalcified cochleas were dehydrated and embedded in paraffin as previously described (Rousset et al., 2020). Mid-modiolar cuts of 5 μm were processed and loaded onto gelatin-coated slides. The Harris’ hematoxylin/eosin staining was performed on five non-consecutive mid-modiolar slides of each cochlea. A Zeiss Axioskop-2, equipped with an AxioCam HRc camera, was used to acquire images from the Rosenthal’s canal corresponding to the basal, medial, and apical turns. Quantification of the spiral ganglion neurons was performed with the open-source software FIJI (ImageJ).

### Image Processing and Analysis for Quantitative Cochlear Cytology

Pictures from cytocochleograms and mid-modiolar cuts were analyzed with the open-source software FIJI (ImageJ). Confocal 10–15-μm z-stack images (0.7 μm steps) were recorded with a 63X objective to quantify the synapses (Ctbp2) in each cochlear turn. The resulting files were reconstructed as two-dimensional images to allow accurate ribbon quantification from the whole sample depth. For each sample section (apical, medial, and basal turns), the number of ribbon synapses of two independent representative areas of 13–15 inner hair cells (IHCs) was analyzed and averaged. Artificial color images were assembled to facilitate analysis and visualization. Hair cell quantification was performed on 20X magnification pictures using the MyoVIIa and 4′,6-diamidino-2-phenylindole markers. IHC and outer hair cell (OHC) populations were counted in two representative areas of 100 μm for each cochlear turn, recording the HC survival average.

The spiral ganglion neuron density was determined by nuclear quantification of Rosenthal’s canal population. Mid-modiolar single z-stack pictures (hematoxylin–eosin) were recorded from five non-consecutive 5-μm sections of each cochlear turn. The number of nuclei was determined manually and normalized to Rosenthal’s canal total area (square millimeter). The measured densities were averaged for the basal, medial, and apical turns.

### Statistics

All data were analyzed using the GraphPad Prism software (version 8.4.3). A two-way analysis of variance test (without repeated measures) followed by Bonferroni multiple comparison correction was used during ABR comparison and histological evaluation (cytocochleograms and synapses). For the comparison of Pt concentration in the liver, kidney, and cochlea by mass spectrometry, Brown–Forsythe and Welch analysis of variance tests with Dunnett’s multiple comparison correction were used. *T*-test unpaired with Welch’s correction was used on the spiral ganglion neuron population comparison. The confidence interval for both statistics was set as 95% (α = 0.05); ^∗^*p* < 0.05, ^∗∗^*p* < 0.01, ^∗∗∗^*p* < 0.005, ^****^p < 0.0005. The least-squares regression approach was used for the Pt/HL correlation and R squared measure.

## Results

### Development of a Preclinical Model of Cisplatin Ototoxicity With Robust Hearing Loss

To develop a preclinical model of cisplatin ototoxicity compatible with the animal welfare legislation in Switzerland, we applied a cisplatin solution (5 mM) directly into the mouse otic bulla through a retroauricular approach (Figure 1). We performed a retroauricular skin incision behind the ear canal (Figure 1A) to access the mice’s middle ear [Figures 1B, C; see also the *Materials and methods* section for a detailed description (*Retroauricular surgical approach*)]. A 25G opening was drilled on the bulla and repeatedly refilled to maintain a constant volume for 15 min (Figure 1D). During the surgery, we observed important variability of the bulla size across animals, and therefore, differences in the middle ear injected cisplatin volume, ranging from 1 to 6 μl (usually 2–4 μl) (Figure 1E). We also observed a correlation between the amount of Pt retained in the cochlea and the volume of cisplatin delivered in the tympanic bulla (*r*^2^ = 0.7517). Based on the volume of cisplatin delivered, we defined two groups of cisplatin-treated animals. Animals receiving a volume of cisplatin <3 μl were classified in the “low cisplatin delivery” group (Figure 1E; blue), whereas animals injected with a volume of cisplatin ≥3 μl were defined as the “high cisplatin delivery” group (Figure 1E; orange). Seven days after the local delivery of cisplatin solution, we observed a significant elevation of click- and pure-tone-evoked hearing thresholds at all tested frequencies in the high cisplatin group (Figure 1F; orange). We also observed significant HL at most tested frequencies (Figure 1; blue) in the low cisplatin group, although the elevation of hearing threshold was overall milder. However, no HL was observed in sham-operated (saline) and non-operated, contralateral ears (Figure 1F; gray and black). Consistently, we observed a significant decrease of the click-evoked ABR wave 1 amplitude only after local delivery of cisplatin (averaging high and low cisplatin data) but not saline (Supplementary Figure 1). We reported comparable ABR threshold shifts 7 and 14 days after local cisplatin delivery, suggesting that the damage induced at D7 is already maximal (Supplementary Figure 2A). However, animals receiving repeated doses of cisplatin had, on average, higher hearing threshold shifts as compared with mice receiving a single local dose of cisplatin (without refilling) (Supplementary Figure 2B).

### Local Delivery of Cisplatin Decreases Hair Cell Numbers in the Mouse Cochlea

**FIGURE 2:**
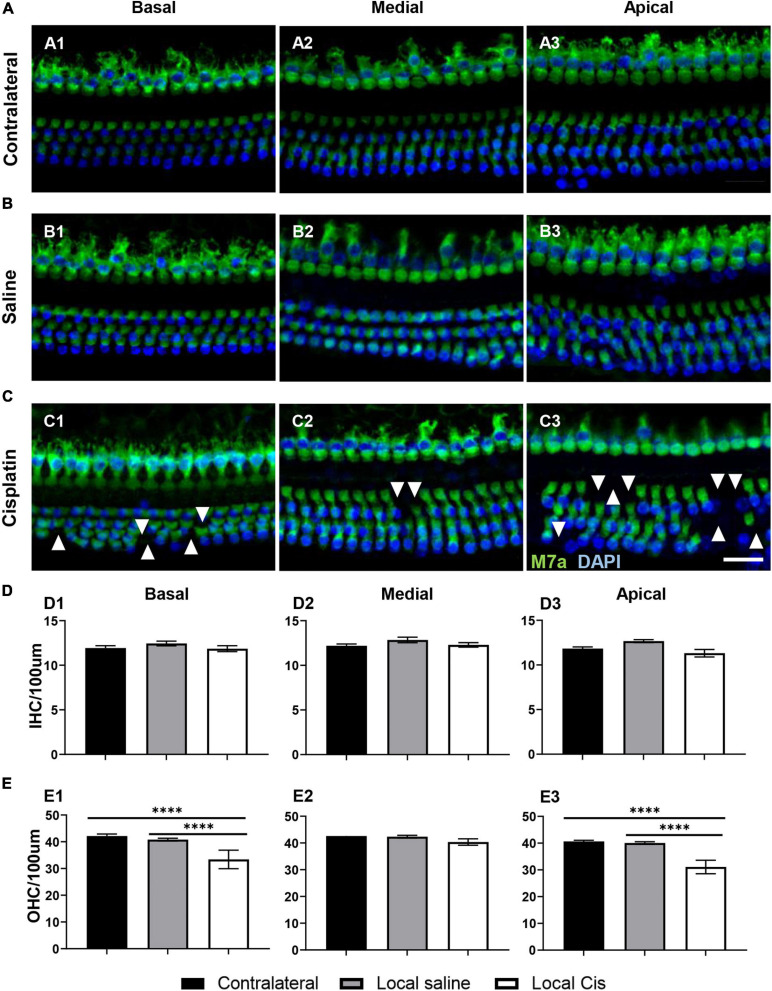
Effects of cisplatin local delivery on IHC and OHC viability. Cytocochleogram showing both IHC and OHC, quantified in a representative area of 100-μm length from basal, medial, and apical cochlear turns, in **(A)** contralateral, **(B)** saline, and **(C)** local-cisplatin (5 mM) cochleae. Samples were stained for 4′,6-diamidino-2-phenylindole (blue) and myosin VIIa (green). **(D)** Bar graphs show number of IHC/100 μm area in **(D1)** basal, **(D2)** medial, and **(D3)** apical turns of cochlea (without subgroup distinction). **(E)** Bar graphs show number of OHC/100 μm area in **(E1)** basal, **(E2)** medial, and **(E3)** apical turns of cochlea (without subgroup distinction). White arrows indicate missing hair cells. Scale bar: 20 μm. *n* = 15. The confidence interval for both statistics was set as 95% (*a* = 0.05); ^****^p < 0.0005.

Similar to what is observed in systemic models (Fernandez et al., 2019), local delivery of cisplatin primarily affected the OHC population (Figures 2C, E). However, no significant loss of IHC could be observed after local cisplatin treatment (Figure 2D1), compared with the non-operated contralateral (Figure 2A) or sham-operated (saline) (Figure 2B). The extent of sensory epithelium damages varied from only a few missing OHC on the low cisplatin group (Supplementary Figure 3) to almost complete HC disruption on the high cisplatin group (Supplementary Figure 4). On average, consistent with the ABR’s hearing thresholds, hair cell apoptosis was more prominent in basal (Figures 2C1, E1) and apical turns (Figures 2C3, E3), whereas a milder effect was observed in the medial turn (Figures 2C2, E2). Importantly, no relevant hair cell loss was related to the surgical approach, as the saline group exhibited similar HC numbers comparable with the contralateral or untreated cochleas from the cisplatin group (Figures 2B, D, E).

### Local Delivery of Cisplatin Leads to Auditory Neuropathy

**FIGURE 3:**
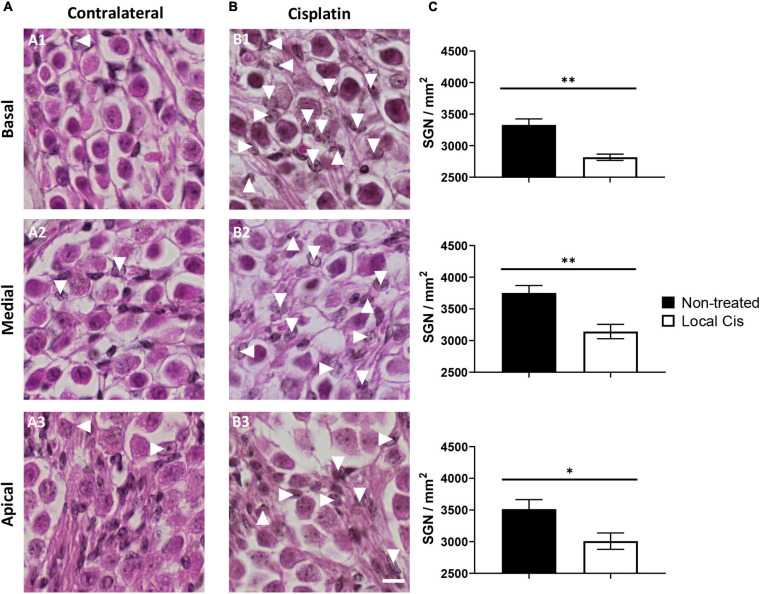
Local delivery of cisplatin effect on auditory neuron viability. **(A,B)** Mid-modiolar histology of mouse cochlea treated with local-cisplatin (5 mM) **(B)** or contralateral cochlea **(A)** showing spiral ganglion. Spiral ganglion neurons in basal **(A1,B1)**, medial **(A2,B2)**, and apical **(A3,B3)** turns were quantified and normalized with size of counting area (in square millimeter). **(C)** Bar graph showing density of auditory neurons. Arrows indicate apoptotic or missing auditory neurons (without subgroup distinction). Scale bar: 10 μm. *n* = 15. The confidence interval for both statistics was set as 95% (*a* = 0.05); ^∗^*p* < 0.05; ^∗∗^*p* < 0.01.

Cisplatin is a potent neurotoxic drug, and loss of auditory neurons (auditory neuropathy) has been previously described in systemic cisplatin mice models (Liu et al., 2019). After local cisplatin delivery, we observed a significant decrease in the auditory neuron density compared with the contralateral cochleae (Figure 3A). Animal receiving “low volume” of cisplatin exhibited only minor loss of auditory neurons and minor morphological changes (Supplementary Figure 3), whereas more pronounced effects could be observed in “high volume” animals (Supplementary Figure 4). Overall, the decrease in the number of auditory neurons was statistically significant in all cochlear turns (Figure 3C), resulting in the presence of apoptotic bodies (Figure 3B, arrows). Therefore, the described local delivery model also recapitulates the cisplatin neurotoxicity observed in conventional systemic models.

### Cisplatin Drastically Decreases the Number of Presynaptic Ribbons

**FIGURE 4:**
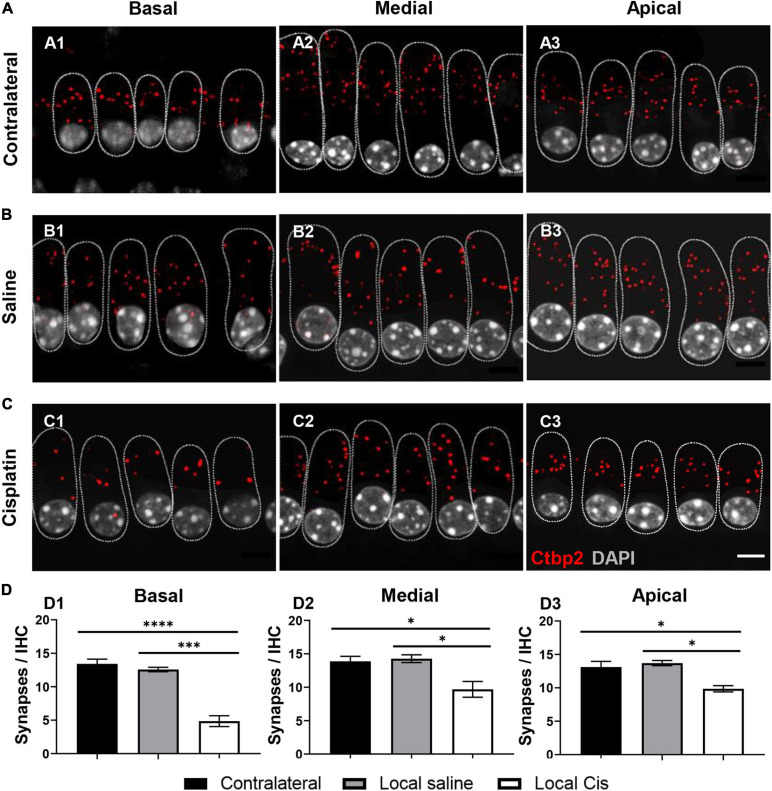
Effect of cisplatin local delivery on number of synapses/IHC. IHC from **(A)** contralateral, **(B)** saline, and **(C)** local-cisplatin (5 mM) cochleas were stained with DAPI (nucleus, white), Ctbp2 (ribbon synapsis, red dots), and myosin VIIa (HCs, substituted by dotted lines to facilitate visualization). **(A1–C1)** Basal, **(A2–C2)** medial, and **(A3–C3)** apical cochlear turns. **(D)** Bar graphs show number of presynaptic ribbons/IHC in apical **(D1)**, medial **(D2)**, and basal **(D3)** turns of cochlea (without subgroup distinction). MyoVIIa staining was replaced with a dotted line, highlighting IHC area, to facilitate Ctbp2 visualization. Scale bar: 5 μm. *n* = 11. The confidence interval for both statistics was set as 95% (*a* = 0.05); ^∗^*p* < 0.05; ^∗∗∗^*p* < 0.005; ^****^p < 0.0005.

The number of auditory neurons synapsing with a single IHC is critical for hearing perception in noisy environments (Kujawa and Liberman, 2015). The stronger effect of local cisplatin treatment was observed on the auditory synapse (Figure 4). Indeed, both animals receiving local low and high volumes of cisplatin exhibited a dramatic decrease in the CtBP2 positive signal for presynaptic vesicles (Supplementary Figures 3, 4). Remarkably, even animals receiving a low amount of cisplatin and where no sensory cell loss could be seen, exhibited a reduced number of auditory synapses, with a prominent effect on the basal and apical turns (Supplementary Figure 3). These data suggest that the auditory synapse is the most vulnerable part of the cochlea with respect to cisplatin ototoxicity and are consistent with the reported amplitude decrease of the ABR wave I (Supplementary Figure 1). Overall, cisplatin-treated cochleae showed a dramatic decrease in the number of synapses compared with the contralateral and saline conditions. This synaptic loss was significant in the basal, medial, and apical turns (Figures 4C1–C3,D) compared with the non-operated contralateral ear (Figure 4A) and with the sham-operated animals (Figure 4B).

### Absence of Systemic Recirculation and Associated Morbidity After Cisplatin Local Delivery

**FIGURE 5:**
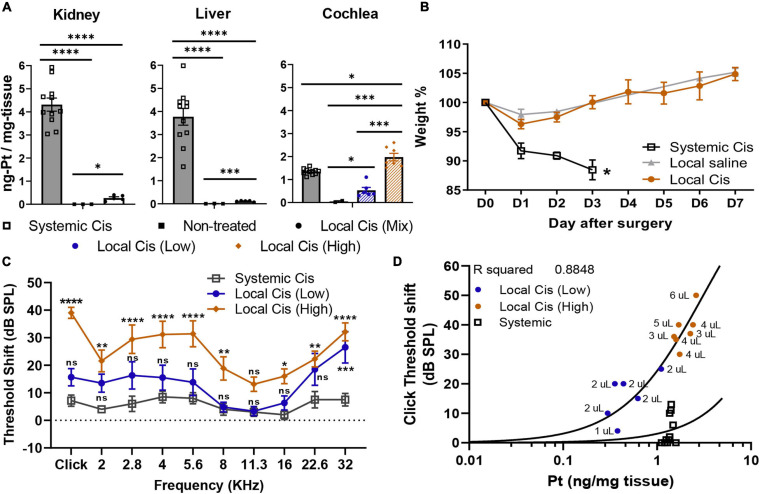
Comparison of systemic *vs*. local cisplatin delivery. **(A)** Comparison of platinum concentration (ng-Pt/mg-tissue) (*n* = 26) in kidney, liver, and cochlea of control (non-injected), IP (10 mg/kg) cisplatin-injected, and cisplatin (5 mM) trans bulla-delivered mice. Mice delivered with cisplatin retroauricularly were divided into two groups according to volume delivered. Group receiving ≥3 μl of cisplatin solution is denominated as “high” cisplatin (orange), and group receiving <3 μl is “low” cisplatin (blue). **(B)** Posttreatment weight evolution. Systemic delivery of 10 mg/kg cisplatin led to weight loss > 10% and early sacrifice at D3, whereas local delivery only mildly affected weight (<5%) in first 2 days post-intervention. Presurgery weight (D0) was set as 100%. **(C)** Local delivery permanent threshold shift dataset (from Figure 1), as calculated from unilateral auditory brainstem response (ABR), in “high” (orange) and “low” (blue) volume cisplatin-treated animals (*n* = 13), is reused for comparison with hearing threshold shifts of animals receiving systemic cisplatin delivery (10 mg/kg). **(D)** Correlation plot between click-evoked hearing threshold shifts and Pt titer 7 days after local cisplatin delivery [5-mM cisplatin (*n* = 13)] and 3 days after systemic delivery (IP 10 mg/kg). Associated to each dot, bulla volume value is displayed (microliters). The confidence interval for both statistics was set as 95% (*a* = 0.05); ^∗^*p* < 0.05; ^∗∗^*p* < 0.01; ^∗∗∗^*p* < 0.005; ^****^p < 0.0005.

Cisplatin accumulation in the liver and kidney is the main cause leading to the systemic toxicity observed in previously established models (Breglio et al., 2017). The cisplatin local delivery model was compared with the systemic model (IP, 10 mg/kg) with respect to general welfare (as assessed by weight measurement and score sheet) and in respect to the Pt retention in the cochlea, kidney, and liver (Figure 5). Animals injected IP with 10 mg/kg of cisplatin exhibited important accumulation of Pt in the kidney, liver, and cochlea (Figure 5A). Seven days after local delivery of cisplatin, we reported a strong Pt accumulation in cochleae of the high cisplatin group, above the levels observed on the systemic delivery, and a significant accumulation on the low cisplatin group (below the systemic levels); however, no significant Pt amount was detected in liver and kidney. Importantly, we did not observe any signs of systemic toxicity such as piloerection, ataxia, or any distress behavior in operated animals (not shown). Consistently, no weight differences were observed between the saline and cisplatin groups, and all the animals significantly overcame the presurgery weight by day 7, without any supplementary diet (Figure 5B). In contrast, mice receiving 10 mg/kg of cisplatin IP (which is usually lower than most of the systemic protocols) lost approximately 15% of weight after only 3 days, leading to premature sacrifice. Unfortunately, in the systemic model, no significant effect of cisplatin on hearing could be observed 3 days after the cisplatin injection (Figure 5C), demonstrating that the cisplatin systemic adverse effects occur before the onset of HL. ABR threshold shifts of local cisplatin delivery, already shown in Figure 1, are also displayed in Figure 5C for the purpose of comparison. In the local cisplatin model, a dose–response curve could be obtained between the amount of Pt retained in the cochlea and the extent of HL, resulting in a robust correlation (*R*^2^ = 0.8848) (Figure 5D). In the systemic model, although a significant amount of Pt was found in the cochlea, no significant HL could be observed, rendering impossible a dose–response comparison. The comparison with the local model is, however, difficult given the premature termination of the experiment at day 3.

## Discussion

A preclinical mouse model of local cisplatin delivery into the otic bulla *via* retroauricular approach has been introduced to study ototoxic HL without relevant systemic adverse effects. The model is relevant, as the main functional and morphological consequences of cisplatin treatment in the cochlea can be robustly induced through local delivery, in line with the ototoxic effects reported after systemic delivery (Sheth et al., 2017). The local delivery model has the main advantage of greatly reducing mortality and morbidity of experimental animals compared with similar studies based on systemic cisplatin delivery, in line with the 3R principles (Parham, 2011; Fernandez et al., 2019). This is not only of relevance for the well-being of the animals, but because more animals survive and are able to live longer, long-term effects of cisplatin on the cochlea can be studied, which has not been possible to this extent in systemic models. In addition, depending on the volume of cisplatin delivered, groups with different severity of HL can be defined and studied, ranging from auditory synaptopathy to sensorineural affection.

In this study, the retroauricular route was chosen in front of the intratympanic delivery procedure, as this approach allows repeated delivery during the same surgical procedure, without increasing the risk of middle ear overpressure and without perforation of the tympanic membrane. Indeed, the retroauricular approach was completely atraumatic on the mice’s hearing. The possibility of conductive HL induced by the surgery was excluded as the sham-operated animal had completely normal hearing (Figure 1F), as was also demonstrated in previous studies with similar approaches (Stevens et al., 2015; Murillo-Cuesta et al., 2017; Rousset et al., 2019).

In the cochlea, the hallmarks of cisplatin ototoxicity can be evoked after local cisplatin treatment, namely predominant outer hair cell loss, loss of auditory neurons, and robust HL. In addition, the more recently discovered loss of synaptic connections between inner hair cells and auditory neurons can be reliably induced. This is of relevance for modeling another typical outcome in cisplatin-treated patients, the hidden HL phenomenon, cursing with tinnitus, and decreased speech perception in the presence of only mild, or absent, measurable HL (El Charif et al., 2019). By reducing the dose of local cisplatin delivered, a hidden HL situation can be adequately modeled through local delivery (Supplementary Figure 3).

Cisplatin has a predominant effect on the patient’s high frequencies (Liberman et al., 2013). This pattern of HL is generally well reproduced on current systemic injected animal models (Fernandez et al., 2019). In the local delivery model, the hearing threshold elevation, as well as the histological features, was also markedly enhanced in the basal cochlear turn in both local cisplatin subgroups, showing similar threshold shifts between low and high volume delivered. These results reinforce the current hypothesis, suggesting this cochlear area is more susceptible to damages even with low doses of cisplatin (Arora et al., 2009). However, the cisplatin local delivery effects were not only limited to high frequencies. Indeed, in the high cisplatin group, significant elevation of the hearing thresholds was observed all along the tonotopic axis (Figures 1F, 5C). By comparison, the systemic cisplatin group exhibited evident signs of morbidity without significant effect on hearing and resulted in the premature sacrifice of the animals on day 3 (Figure 5C).

Differences in the pattern of HL observed between systemic and local delivery models (Figure 5C) could be explained by the cisplatin entry route. The stria vascularis could be the main entry point for systemic cisplatin delivery (through the vasculature) (Karasawa and Steyger, 2015). However, in the local delivery model, the mass spectrometry data showed only residual amounts of Pt in the analyzed organs compared with the systemic approach, including the liver, kidney, or even in the contralateral ear (Figure 5A), excluding important cisplatin diffusion through the vasculature. Most likely, cisplatin delivered locally predominantly diffuses from the otic bulla directly into the cochlea through the round window membrane or directly reaching the modiolus through the cochlear bone (Mikulec et al., 2009). Direct diffusion of cisplatin to the modiolus is indeed an interesting hypothesis that could explain the predominant effect on auditory neurons and the relatively milder effect on the sensory epithelium but requires further validation. Nevertheless, the pattern of HL observed on the systemic group (Figure 5C), although significantly lower, showed similarities with the local model, with stronger effects on the 22.6–32 kHz frequencies, but also on the tested frequencies between 2.8 and 5.6 kHz.

Although all animals in the 5-mM cisplatin local delivery group were injected following the same exact protocol (see *Materials and methods*), we observed differences in the Pt (cisplatin) retention in the cochlea between animals. Initially, we correlated this apparent high variability with the animal sex. However, we did not observe such sex difference in the Pt cochlear concentration at D3 after the systemic injection (data not shown), excluding a sex-related protection mechanism hypothesis, previously suggested (Park et al., 2019). Nevertheless, we believed that anatomical variations, as previously reported for the human cochlea (Braga et al., 2019), could explain the different cisplatin concentrations observed between animals. Accordingly, we observed a correlation between the tympanic bulla volume capacity (size) and the cochlear Pt concentration (*R*^2^ = 0.7517) (Figure 1E) and thus the extent of HL (*R*^2^ = 0.8848) (Figure 5D), explaining the observed variability. This intrinsic variability allowed us to recapitulate the effect of low and high cisplatin doses on the hearing system.

Cisplatin concentrations in the cochlea were slightly superior when delivered through the local delivery approach, on the high local group, compared with the systemic route (Figure 5A). However, the difference in the HL incidence between groups was rather considerable (Figure 5C). However, we must be cautious with the interpretations of these results, as IP injected animals already lost 15% of their weight 72 h after the injection and were immediately sacrificed. The animals in the local delivery groups (low and high volume delivered) were sacrificed 1 week after the injection, at the end of the experimental design. In the systemic group, the observed effect on the hearing thresholds was probably still incomplete due to the early sacrifice time (D3), rendering a direct comparison between both models difficult. Alternatively, these differences could be explained following a different penetration profile of cisplatin in the inner ear when delivered locally or systemically (see above).

Interestingly, following the different cisplatin (Pt) fixation rates depending on the bulla size, we observed a strong correlation (*R*^2^ = 0.8848) between the Pt concentration retained in the cochlea and the hearing threshold shift (click threshold, Figure 5D). Hence, from the extent of HL observed, our model allows accurate prediction of the Pt concentration retained in the cochlea. Such a correlation is important and demonstrated that even when low doses of cisplatin are retained in the cochlea, a significant effect on the animal hearing is observed. Similarly, a correlation plot comparison representing HL and Pt could be used to identify new therapeutical targets following local delivery on knockout models. This approach can be implemented to preliminarily evaluate candidate targets, reducing the number of animals deployed for systemic evaluation and therefore reducing the associated morbidity of these studies, in line with the 3R principles.

## Conclusion

The presented preclinical model of local delivery of cisplatin to the cochlea adequately models the known changes in inner ear morphology and function after the ototoxic insult without inducing relevant morbidity, in line with 3R principles. In numerous countries, animal welfare regulation is not compatible with current systemic cisplatin preclinical models, which generally lead to important loss of weight, morbidity, and mortality before reaching significant ototoxic effects. Due to the unchanged life expectancy of locally treated animals, the long-term effects of cisplatin ototoxicity can now be adequately addressed. Furthermore, local delivery HL–Pt correlation comparison can be used to preliminary evaluate new therapeutic targets, significantly reducing the animals intended for systemic cisplatin evaluation. Taken together, the local delivery model offers many advantages and will, at least in our laboratory, be used to identify genetic targets for drug-based otoprotection and to further evaluate the possibility of gene therapy-based prevention of cisplatin-induced HL in preclinical studies (Waissbluth et al., 2012).

## Data Availability Statement

The raw data supporting the conclusions of this article will be made available upon request by the authors.

## Ethics Statement

The animal study was reviewed and approved by local veterinary office and the Commission for Animal Experimentation of the Canton of Geneva (authorization number GE/149/18).

## Author Contributions

FR, PS, and K-HK conceived the project. GN-S, PS, and FR conceived and planned the experiments. GN-S carried out the experiments and surgeries. GN-S analyzed the data. FR supervised the data curation. GN-S and FR drafted the manuscript and designed the figures. PS finalized the manuscript writing. SL carried out the mass spectrometry analysis. MC worked out the technical details and sample preparation. AT supervised the mass spectrometry analysis. FV contributed to the project design. All authors discussed the results and contributed to the final manuscript.

## Conflict of Interest

The authors declare that the research was conducted in the absence of any commercial or financial relationships that could be construed as a potential conflict of interest.
