# Supplementary material for: Local Cisplatin Delivery in Mouse Reliably Models Sensorineural Ototoxicity Without Systemic Adverse Effects
Source: Front Cell Neurosci. 2021 Jul 14;15:701783. doi: 10.3389/fncel.2021.701783 (PMC8316727; doi:10.3389/fncel.2021.701783)
Supplement: Supplementary file 1 [file Data_Sheet_1.PDF]

Supplementary figure 1:

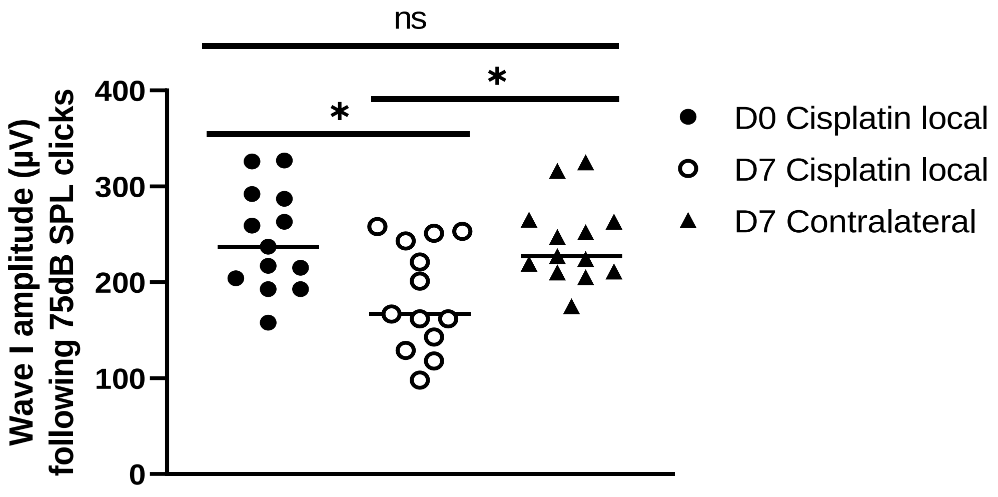

Supplementary figure 1: Clicks-evoked ABR wave I amplitude following local cisplatin treatment. Unilateral wave I ABR amplitude as generated in response to 75 dB SPL clicks on the same ear before cisplatin local administration (D0) or 7 days after treatment (D7 cisplatin local) and on the contralateral ear (D7 contralateral).

Supplementary figure 2:

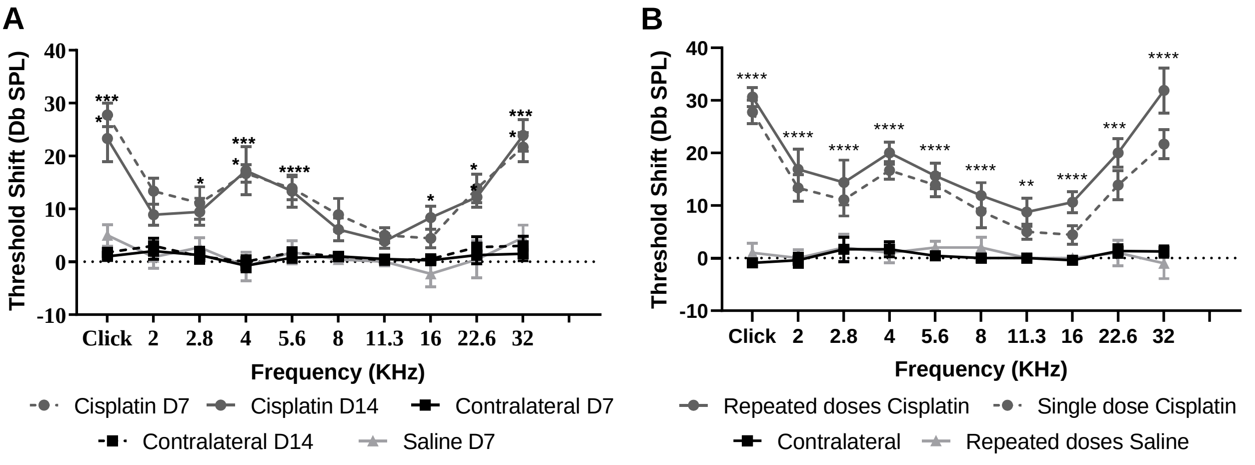

Supplementary figure 2: Optimization of time and dose of cisplatin intra-bulla delivery. (**A**) Effect of the time of treatment with a single intra-bulla delivery (2 µL) of cisplatin solution (1mg/mL), ABR hearing thresholds were recorded 7 and 14 days following cisplatin or saline delivery. Threshold shifts were obtained by substraction of the pre ABR value (D0). (**B**) Single vs. repeated intra-bulla injection of cisplatin or saline in the mouse tympanic bulla. Protocols based on single or repeated intra-bulla dose delivery (8 doses of 2 µL during 15 min of treatment) were compared on hearing threshold shift as determined by ABR measurement

Supplementary figure 3:

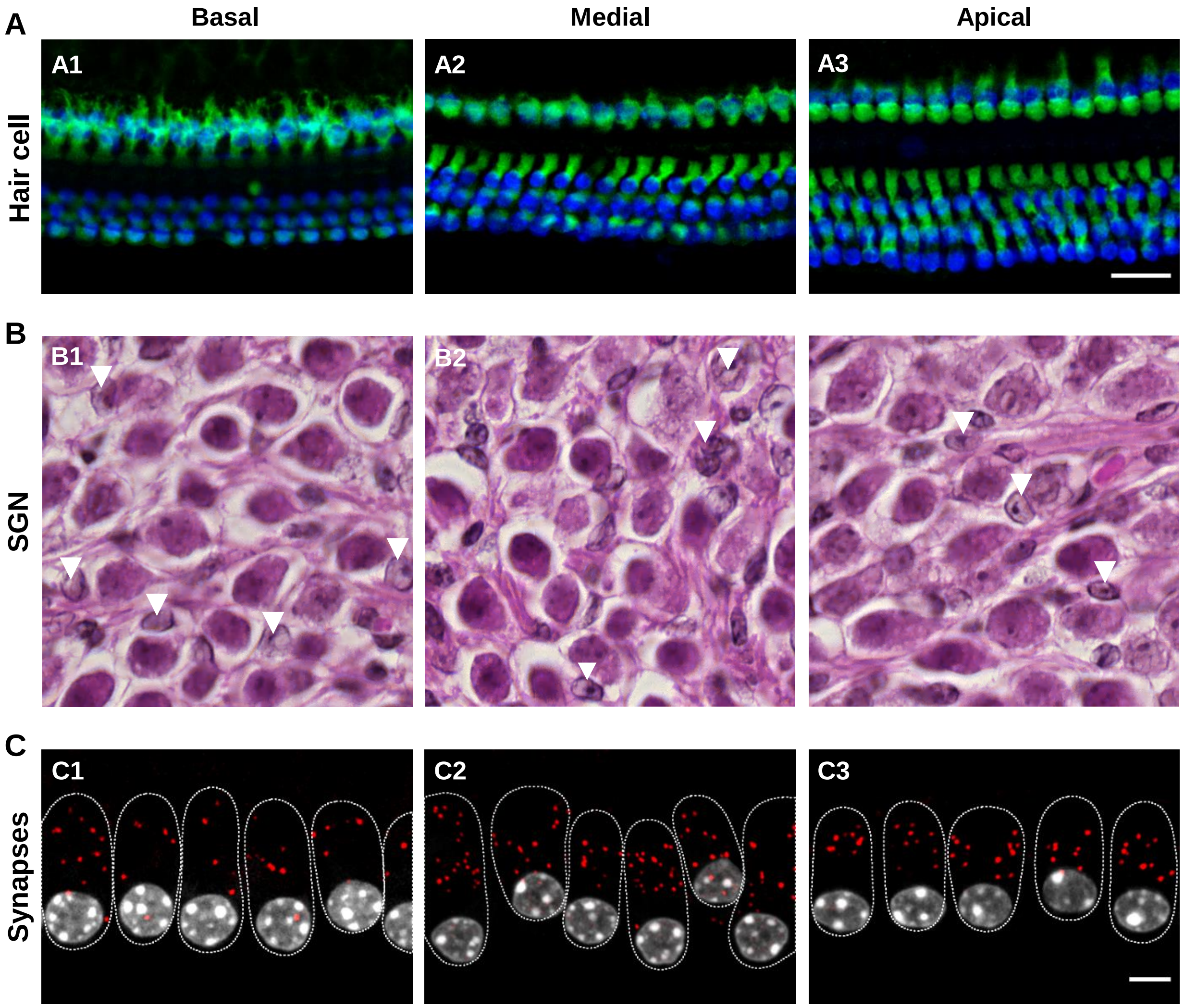

Supplementary figure 3: Histologic defects resulting from low cisplatin volume delivery. Representative histological images from the "low" cisplatin volume delivery sub-group were grouped in order to promote qualitative evaluation of the effects from low cisplatin fixation on the cochlea physiology. (**A**) Cytocochleograms were stained for DAPI (blue) and myosin VIIa (green). (**B**) Mid-modiolar histology was counterstained following haematoxylin-eosin protocol, showing apoptotic cells (white arrows). (**C**) IHC were stained with DAPI (nucleus, white), Ctbp2 (ribbon synapsis, red dots) and myosin VIIa (dotted line). Scale bar A: 20 um. Scale bar B: 10 um. Scale bar C: 5 um.

Supplementary figure 4:

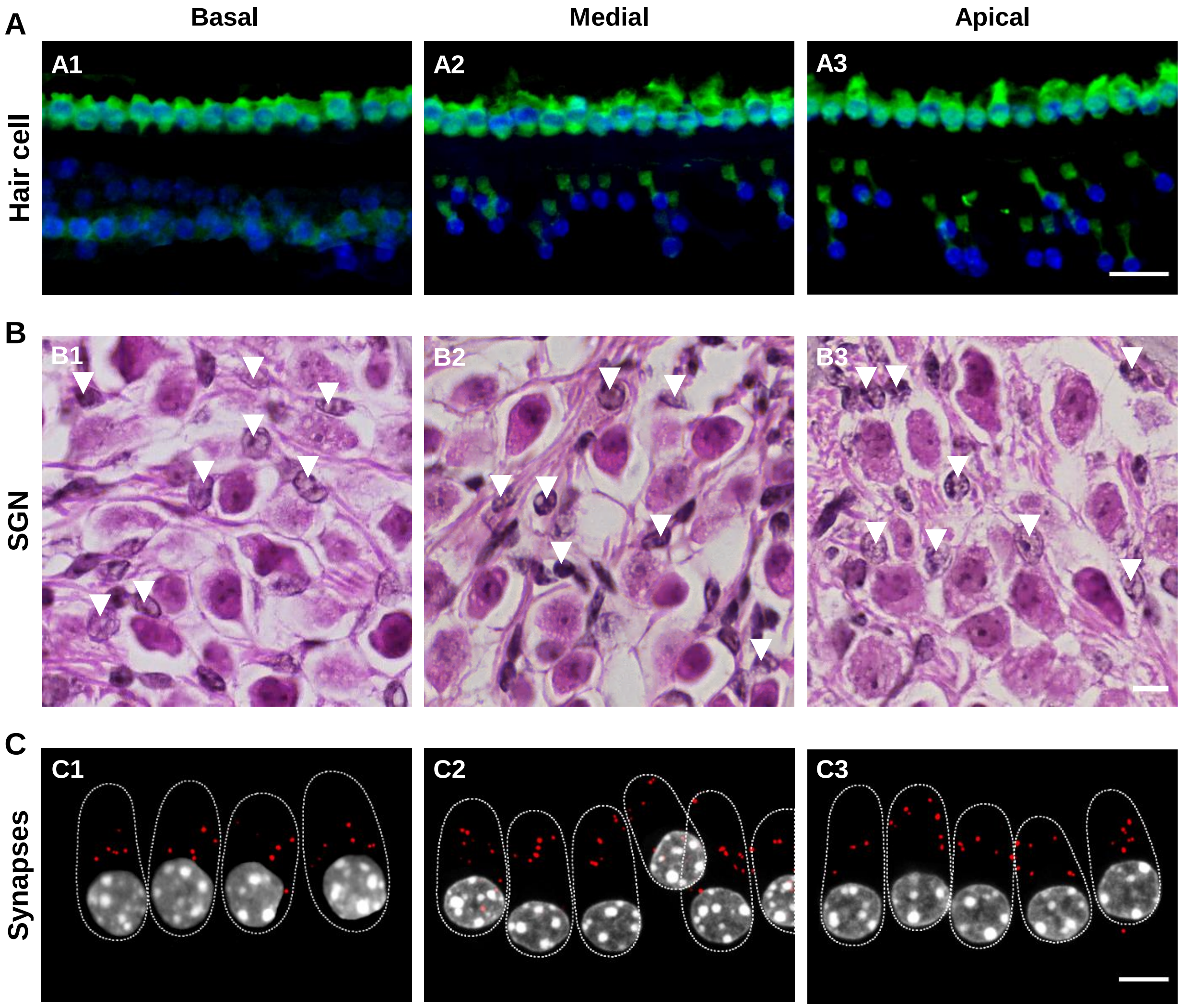

Supplementary figure 4: Histologic defects resulting from high cisplatin volume delivery. Representative histological images from the "high" cisplatin volume delivery sub-group were grouped in order to promote qualitative evaluation of the effects from high cisplatin fixation on the cochlea physiology. (**A**) Cytocochleograms were stained for DAPI (blue) and myosin VIIa (green). (**B**) Mid-modiolar histology was counterstained following haematoxylin-eosin protocol, showing apoptotic cells (white arrows). (**C**) IHC were stained with DAPI (nucleus, white), Ctbp2 (ribbon synapsis, red dots) and myosin VIIa (dotted line). Scale bar A: 20 um. Scale bar B: 10 um. Scale bar C: 5 um.
